# Association of socio-economic deprivation with COVID-19 incidence and fatality during the first wave of the pandemic in Italy: lessons learned from a local register-based study

**DOI:** 10.1186/s12942-023-00332-9

**Published:** 2023-05-04

**Authors:** Francesca Fortunato, Roberto Lillini, Domenico Martinelli, Giuseppina Iannelli, Leonardo Ascatigno, Georgia Casanova, Pier Luigi Lopalco, Rosa Prato

**Affiliations:** 1 Hygiene Unit, Policlinico Foggia Hospital, Department of Medical and Surgical Sciences, University of Foggia, Foggia, Italy; 2 Analytical Epidemiology & Health Impact Unit, Fondazione IRCCS Istituto Nazionale dei Tumori, Milan, Italy; 3 IRCCS-INRCA National Institute of Health & Science on Ageing, Centre for Socio-Economic Research on Ageing, Ancona, Italy; 4 Department of Biological and Environmental Sciences and Technology, University of Salento, Lecce, Italy

**Keywords:** COVID-19, Health inequalities, Deprivation index, Incidence, Mortality, Socio-economic status, Socio-economic and socio‑demographic factors

## Abstract

**Background:**

COVID-19 has been characterised by its global and rapid spread, with high infection, hospitalisation, and mortality rates worldwide. However, the course of the pandemic showed differences in chronology and intensity in different geographical areas and countries, probably due to a multitude of factors. Among these, socio-economic deprivation has been supposed to play a substantial role, although available evidence is not fully in agreement. Our study aimed to assess incidence and fatality rates of COVID-19 across the levels of socio-economic deprivation during the first epidemic wave (March–May 2020) in the Italian Province of Foggia, Apulia Region.

**Methods:**

Based on the data of the regional active surveillance platform, we performed a retrospective epidemiological study among all COVID-19 confirmed cases that occurred in the Apulian District of Foggia, Italy, from March 1st to May 5th, 2020. Geocoded addresses were linked to the individual Census Tract (CT) of residence. Effects of socio-economic condition were calculated by means of the Socio-Economic and Health-related Deprivation Index (SEHDI) on COVID-19 incidence and fatality.

**Results:**

Of the 1054 confirmed COVID-19 cases, 537 (50.9%) were men, 682 (64.7%) were 0–64 years old, and 338 (32.1%) had pre-existing comorbidities. COVID-19 incidence was higher in the less deprived areas (p < 0.05), independently on age. The level of socio-economic deprivation did not show a significant impact on the vital status, while a higher fatality was observed in male cases (p < 0.001), cases > 65 years (p < 0.001), cases having a connection with a nursing home (p < 0.05) or having at least 1 comorbidity (p < 0.001). On the other hand, a significant protection for healthcare workers was apparent (p < 0.001).

**Conclusions:**

Our findings show that deprivation alone does not affect COVID-19 incidence and fatality burden, suggesting that the burden of disease is driven by a complexity of factors not yet fully understood. Better knowledge is needed to identify subgroups at higher risk and implement effective preventive strategies.

## Background

The Coronavirus Disease 2019 (COVID-19) pandemic has caused substantial morbidity and mortality worldwide. As of June 1st 2020, 233,607 cases of confirmed Severe Acute Respiratory Syndrome coronavirus 2 (SARS-COV-2) infection were reported in Italy with a case fatality rate of 13.8% [1].

Despite the widespread opinion that the novel coronavirus has not discriminated, SARS-CoV-2 infection, like many other infectious diseases, seems to have affected predominantly the portion of the population in socio-economic disadvantage. Indeed, many studies have demonstrated disproportionate morbidity and mortality among socioeconomically disadvantaged population groups with higher hospitalisation and death rates among neighbourhoods with the highest proportion of ethnic minorities and individuals living in poverty [2–14]. On the other hand, a minority of studies did not show these associations or even reported conflicting results [15–20].

Several factors may have contributed to the observed inequality including the level of education, the overrepresentation of minorities in public-facing occupations, overcrowded living and working conditions, inappropriate information, and prevalence of pro-inflammatory unhealthy lifestyle [21–24]. All these factors negatively affect the overall health conditions and are correlated with an increased risk of several diseases such as cardiovascular diseases, diabetes, chronic respiratory diseases, and some cancers, which in turn may have increased COVID-19 incidence and mortality risk [25, 26]. The extent to which these disparities have been related to socio-economic versus biological factors still needs further understanding [4, 27]. Indeed, the knowledge gathered during the course of the pandemic investigated the complexity of the factors involved and distinguished four potential pathways: vulnerability (the burden of pre-existing health conditions), susceptibility (the *status* of the immune function), level of exposure (e.g., working or travelling conditions), and risk of transmission (e.g., due to crowded housing) [28].

The analysis of the Italian National Institute of Statistics (Istat) on the effect of socio-economic disparities in COVID-19 mortality demonstrated that during the first quarter of 2020, alongside an overall increase of the crude mortality rate, a more pronounced increase in mortality rate was observed among the most disadvantaged groups [25]. Istat data collected during the successive waves confirm the amplification effect of inequalities ascribable to the pandemic and its negative impact on mortality [26, 29]. A study conducted in the Emilia Romagna Region confirmed that people living in the most disadvantaged census block were exposed to an increased risk of both overall and COVID-19-related mortality during the first epidemic peak [30].

In such context, collection and analysis of socio-economic data appeared necessary to examine a potential disproportionate burden of COVID-19 across the different population subgroups. Deprivation indexes are useful measures for health inequalities analysis by identifying and evaluating the relationships between socio-economic status (SES) and health conditions. These indexes usually refer to geographical aggregations and are used as a proxy of the individual’s conditions according to the area of residence [31, 32].

By using deprivation indexes, our study aimed to assess the impact of socio-economic deprivation on the incidence and fatality of COVID-19 during the first epidemic wave (March–May 2020) in the Italian Province of Foggia, Apulia Region.

## Methods

This study involved all the 1054 cases laboratory confirmed as SARS-CoV-2 positive by real-time RT-PCR, regardless of the presence of symptoms or patient management (hospitalised or managed at home), that were recorded in the Foggia province (Apulia region, Southern Italy) from March 1st to May 5th, 2020. The cases were selected and followed-up for the same period, as previously described [33, 34].

In particular, the following case characteristics were considered: sex, age, health status at the end of the follow-up, and presence/absence of at least one comorbidity (specifically: cancer, diabetes, cardiovascular diseases, HIV infection, chronic lung diseases, kidney diseases, other metabolic diseases, obesity two subgroups: BMI between 30 and 40 kg/m^2^ or BMI > 40 kg/m^2^, liver diseases, neurological diseases, other diseases).

Additionally, the belonging to the healthcare personnel of public or private healthcare system and/or the connection with a nursing home (both as a patient or a care worker) were also recorded.

**Fig. 1:**
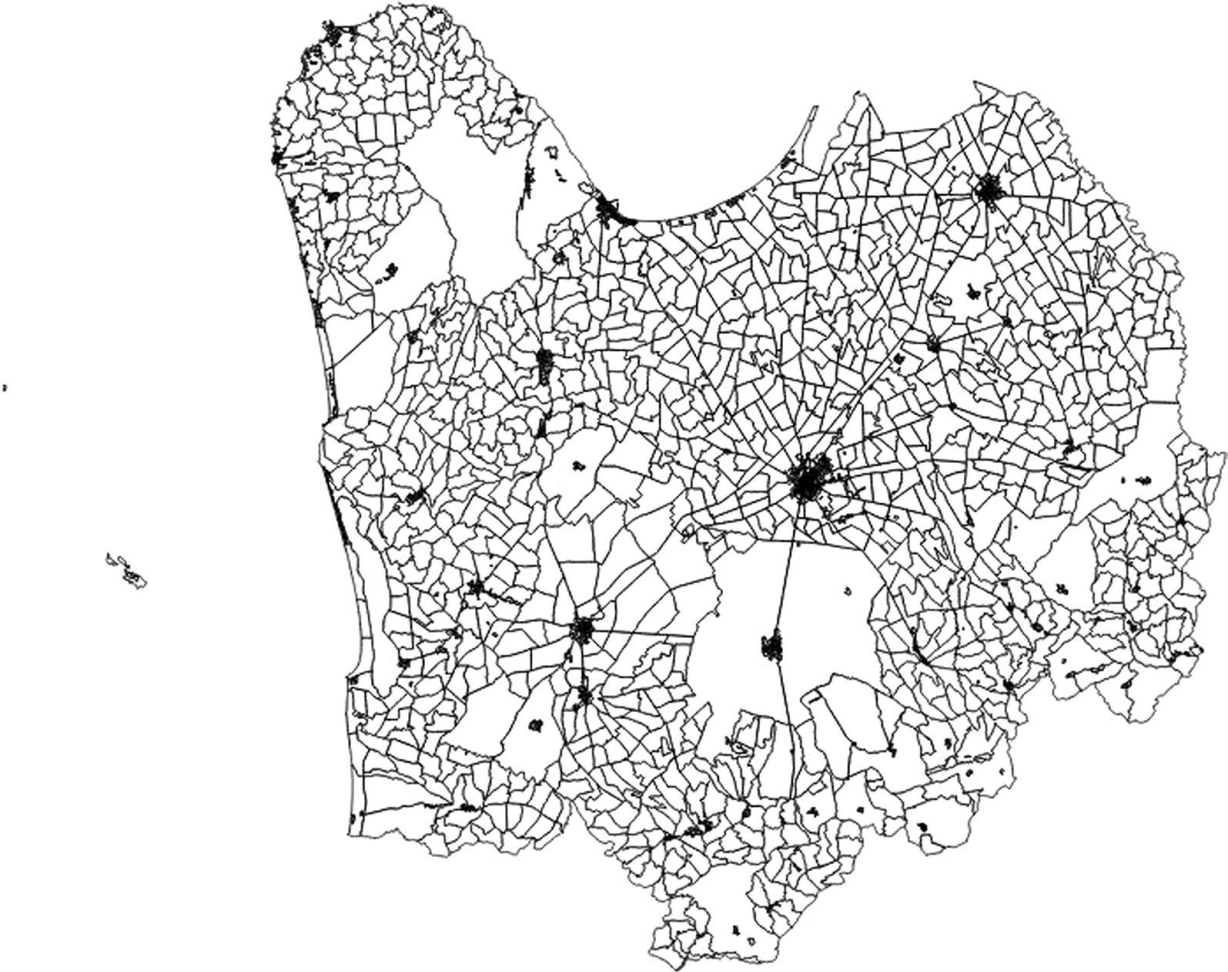
Census Tracts of the Foggia province

In order to attribute to every case his/her Census Tract (CT) of residence (referred to the 2011 Population and Housing National Census [35]), the individual complete residence address and X&Y Cartesian coordinates were collected and checked by the Geographic information system (GIS) procedure [32, 36, 37] (Fig. 1).

**Fig. 2:**
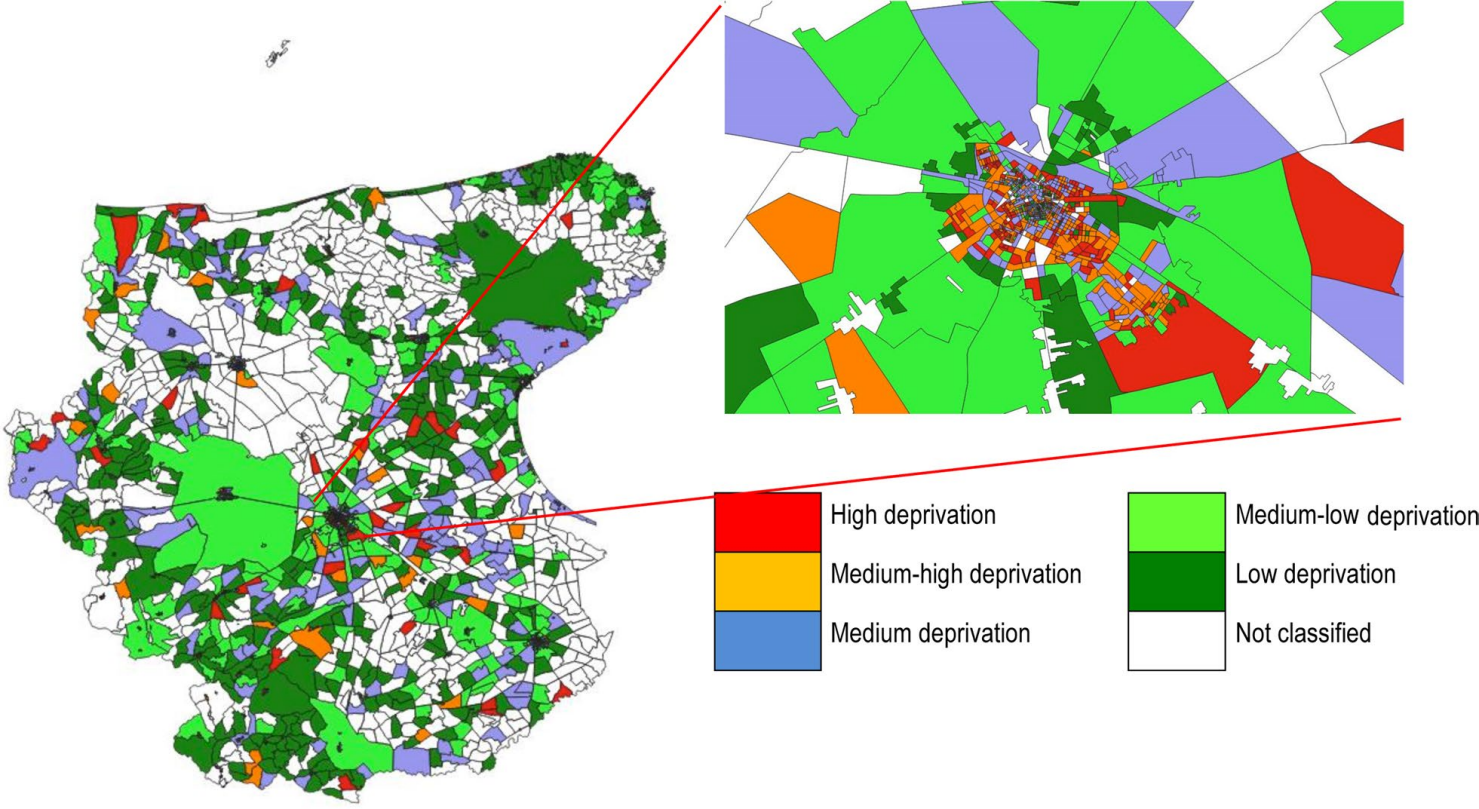
Census Tracts of the Foggia province and the Foggia municipality (zoom) by SEHDI groups

Information on the socio-economic condition of the cases was computed at CT level for the Foggia province and the Socio-Economic and Health-related Deprivation Index (SEHDI) was calculated as previously described [32, 38]. In particular, the SEHDI is able to highlight the relevance of the social aspects of deprivation (e.g., older age, availability of familial network and social support, etc.) when considering how a population cope with the disease spreading, prevention, or patient care, considering the peculiarities of the local area where it is calculated. We set the older age threshold at age 65 based on socioeconomic considerations (e.g., health status, retirement age) in line with previous published studies [32, 38]. Factors and variables composing the SEHDI 2011 are presented in Table 1 and Fig. 2. Due to some errors in the geographical data of 10 cases, the SEHDI score and classification were attributed to 1,044 cases (99.1% of all cases).

**Table 1:** Factors and variables^1^ composing the SEHDI 2011. Old-age index, age, sex distribution by deprivation groups

Explained variance and composing variables by factor			
Factor 1 = 28.8%	Factor 2 = 15.9%	Factor 3 = 15.4%	Factor 4 = 14.1%
% single-member families aged 65 +	Average N of people per family	Old-age index	% separated & divorced
% widowed	% houses with bath or shower	% primary school or less	
% single-member families			

Total explained variance = 74.2%													
Groups SEHDI 2011	0–64 years						65 + years						Old-age Index
	M + F	% M + F	M	% M	F	% F	M + F	% M + F	M	% M	F	% F	
High deprivation	27,072	5.3	13,227	5.2	13,845	5.5	11,932	10.1	4776	9.4	7156	10.7	353.9
Medium–high deprivation	94,337	18.6	46,891	18.4	47,446	18.7	33,804	28.7	13,681	26.9	20,123	30.1	225.7
Medium deprivation	239,111	47.0	119,341	46.9	119,770	47.2	57,076	48.4	24,920	49.0	32,156	48.0	125.8
Medium–low deprivation	114,319	22.5	57,623	22.7	56,696	22.3	12,601	10.7	6240	12.3	6361	9.5	57.5
Low deprivation	33,400	6.6	17,193	6.8	16,207	6.4	2420	2.1	1270	2.5	1150	1.7	16.8
All population	508,239		254,275		253,964		117,833		50,887		66,946		127.21 (L)

*L* = *statistically significant linear trend*

The SEHDI was computed as a score from 0 (lowest deprivation) to 100 (highest deprivation) attributed to all population and re-classified in 5 groups (from 1 = High deprivation to 5 = Low deprivation), in which the population has been normally distributed across the groups, according to the Agnelli-Cadeiras algorithm [32, 39].

The incidence of COVID-19 was examined by Standardised Incidence Ratio (SIR; SIRs were standardised on the 2011 overall provincial population [40]). The effects of the socio-economic deprivation on the incidence were analysed by Analysis of Variance (ANOVA) of the SIRs trend through the 5 deprivation groups (F test with statistical significance threshold at p < 0.05) [41].

The vital status (alive/dead at the end of follow-up) by personal characteristics and comorbidities was examined by two-way tables, considering the percentage by row and computing the χ^2^ as connection index (statistical significance at p < 0.05) [41]. The potential effects of the individual characteristics and classification by SEHDI on the vital status at the end of follow-up were analysed by multilevel mixed-effects logistic regression model (vital status as dependent variable, personal characteristics as individual fixed-effects covariates, SEHDI groups as the area random-effects covariates). The reliability and statistical significance of the model were tested by log likelihood ratio and comparison with χ^2^ (statistical significance at p < 0.05) [41]. All the procedures (GIS and statistical analyses) were performed by QGIS 3.10 and Stata 14.0 software.

## Results

Table 1 reports the census variables composing the SEHDI factors, the variance explained by each factor, and the total explained variance. The distribution of old-age index and the distribution by age groups, sex and deprivation clusters individuated by the SEHDI 2011 are also reported. The SEHDI factors pertained to the domains which are classically recognised as linked to the socio-economic status of a population: the family structure, the household characteristics, and the education level. In terms of family structure (factor 1), it was included variables highlighting the difficulties of single-person households, particularly if composed by an elderly individual living alone. Furthermore, factor 2 described the dwelling conditions (i.e., the percentage of houses with a bath or a shower), factor 3 emphasised the association between deprivation and low educational level, while factor 4 considered the percentage of separated and divorced persons. The analysed population of Foggia province (n = 626,072) was quite young (the 81.2% was younger than 65 years). Among the elderly, the percentage of women was slightly prevalent than that of men (n = 66,946, 10.7% vs. n = 50,887, 8.1%). The population distribution by deprivation groups differed in age and sex: lower deprivation was associated with younger age while higher deprivation was more frequently observed in the elderly, mostly in women. The old-age index distribution showed a statistically significant positive linear trend (values increased as deprivation grew).

Figure 2 represents the distribution of CTs by SEHDI groups in the Foggia province and in the capital city (Foggia municipality). The presence of high and medium–high deprivation groups was higher within the Foggia municipality and in the proximity of the other main municipalities of the province (Cerignola, Lucera, and San Severo). In Foggia municipality, the proximity and the higher concentration of the most deprived groups were evident, mainly in the Southern part of the city centre.

Table 2 shows the characteristics of COVID-19 cases, along with the distribution by sex, age, and deprivation groups, as a descriptive comparison with the overall province population. Both case distribution and population distribution by sex were well-balanced in the province. On the contrary, the case distribution by age differed from the overall population distribution: the percentage of elderly (≥ 65 years old) among COVID-19 cases was 35.3% compared with 18.6% among the provincial population.

**Table 2:** Comparison of sex, age, deprivation distributions between COVID-19 cases and the overall Foggia province population

COVID-19 case characteristics	N	%	% on overall province population
Sex			
Men	537	50.9	48.8
Women	517	49.1	51.2
Age groups			
0–64 years	682	64.7	81.4
65 + years	372	35.3	18.6
SEHDI 2011 groups			
High deprivation	62	5.9	6.2
Medium–high deprivation	205	19.6	20.5
Medium deprivation	406	38.9	47.3
Medium–low deprivation	275	26.3	20.3
Low deprivation	96	9.2	5.7
Employed in the health system			
No	760	72.1	
Yes	294	27.9	
Connection with a nursing home			
No	877	83.2	
Yes	177	16.8	
Vital status			
Alive	924	87.7	
Dead	130	12.3	

Additionally, differences were observed in the distributions across deprivation groups: the majority of COVID-19 cases were resident in the least deprived areas, while the provincial population was distributed on a Gaussian basis by methodological definition. Moreover, among COVID-19 cases the percentage of individuals having a connection with nursing facilities was low (16.8%) while the percentage of healthcare system employees was slightly higher (27.9%). Lastly, the case fatality rate was 12.3%.

Table 3 provides a more complete description of the COVID-19 case group: 338 of 1054 cases (32.1%) had pre-existing comorbidities, most of which were significantly linked to ageing (p < 0.05; cancer, diabetes, cardiovascular disease, chronic lung diseases, kidney diseases, other metabolic diseases, liver diseases, neurological diseases, other diseases), while obesity and HIV infection were not related to ageing. No direct correlation between deprivation and comorbidities was observed.

**Table 3:** Comorbidities in the COVID-19 case group

Variable	N	%	χ2(p) applied to age groups	χ2(p) applied to SEHDI groups
Comorbidity				
No	716	67.9	*p* = 0.000*	*p* = *0.731*
Yes	338	32.1		
Type of comorbidity				
Cancer	36	3.4	*p* = 0.000*	*p* = *0.333*
Diabetes	70	6.6	*p* = 0.000*	*p* = *0.377*
Cardiovascular diseases	219	20.8	*p* = 0.000*	*p* = *0.559*
HIV infection	8	0.8	*p* = *0.382*	*p* = *0.331*
Chronic lung diseases	46	4.4	*p* = 0.002*	*p* = *0.316*
Kidney diseases	33	3.1	*p* = 0.000*	*p* = *0.588*
Other metabolic diseases	14	1.3	*p* = 0.022*	*p* = *0.070*
Obesity:				
BMI between 30 and 40 kg/m^2^	13	1.2	*p* = *0.810*	*p* = *0.824*
BMI > 40 kg/m^2^	10	1.0	*p* = *0.309*	*p* = *0.822*
Liver diseases	12	1.1	*p* = 0.000*	*p* = *0.237*
Neurological diseases	34	3.2	*p* = 0.000*	*p* = *0.133*
Other diseases	53	5.0	*p* = 0.001*	*p* = *0.830*

Foggia province, Apulia Region, Italy

^*^ = statistical significance at p < .05 level

The effects of socio-economic deprivation on the incidence of COVID-19 are presented in Table 4. The distribution of SIRs in the overall population and by sex, age groups, and age by sex were analysed across the five SEHDI clusters. It was confirmed that COVID-19 incidence was higher in the least deprived individuals with a statistically significant negative linear trend (p < 0.05). Similar trends were observed when two different age groups were considered (<65 or  ≥65 years old). When comparing each sex with the age by sex, the same trend was maintained in men regardless of the age group while in the female population the same trend was maintained only in the younger group (0–64 years old) (p < 0.05).

**Table 4:** Standardised Incidence Ratio trends by sex, age, and age by sex across SEHDI groups

SEHDI groups		Standardised Incidence Ratios (on provincial basis)								
		M + F	Sex		Age groups		Men’s age groups		Women’s age groups	
			M	F	0–64	65 +	0–64	65 +	0–64	65 +
High D	Rates	3.67	1.41	2.37	4.12	3.05	1.80	0.70	2.30	2.95
	N of cases	62	31	31	40	22	22	9	18	13
Medium–high D	Rates	4.81	2.32	2.56	5.83	3.69	2.82	1.51	3.06	2.07
	N of cases	205	100	105	120	85	60	40	60	45
Medium D	Rates	5.28	2.75	2.51	6.30	3.99	3.12	1.73	4.43	2.28
	N of cases	406	223	183	253	153	142	81	111	72
Medium–low D	Rates	13.19	7.12	6.06	12.57	12.00	6.67	7.89	5.92	2.92
	N of cases	275	136	139	187	88	89	47	98	41
Low D	Rates	16.78	6.29	5.06	15.41	11.25	6.11	2.18	3.26	11.15
	N of cases	96	44	52	74	22	35	9	39	13
Total	Rates	*8.45*	*3.88*	*3.55*	*8.70*	*6.47*	*4.04*	*2.62*	*3.93*	*4.11*
	N of cases	*1044*	*534*	*510*	*674*	*370*	*348*	*186*	*326*	*184*
P Trend		0.0218 L	0.0364 L	0.0502 N.S	0.0044 L	0.0048 L	0.0057 L	0.0392 L	0.0285 L	0.3417 N.S

Foggia province, Apulia Region, Italy

*D* *deprivation*, *p* *Linearity F test with Bonferroni correction: L* *linear trend, N.S.* *no statistical significance*

The effects of individual characteristics and socio-economic deprivation on the vital status of cases at the end of the follow-up period are shown in Tables 5 and 6. A higher fatality was observed in male cases (15.6% males vs. 8.9% females), elderly cases (31.2% in over-65s vs. 2.1% in under-65s), cases having a connection with a nursing home as guests or staff members (17.5% with connection vs. 11.8% without connection), and cases affected by at least one comorbidity (24% with comorbidities vs. 6.8% without comorbidities) (p < 0.05). No statistically significant effect was associated with the socio-economic status. Significant protection was observed for healthcare workers (16.8% of deaths in non-healthcare workers vs. 0.7% in healthcare workers; p < 0.001).

**Table 5:** Vital status and case characteristics

COVID-19 case characteristics	Overall	Alive		Dead		Pearson χ2	*p*
	N	N	%	N	%		
Sex							
Men	537	453	84.4	84	15.6	11.1	0.001
Women	517	471	91.1	46	8.9		
Age groups							
0–64 years	682	668	98.0	14	2.1	188.9	0.000
65 + years	372	256	68.8	116	31.2		
SEHDI 2011 groups							
High deprivation	62	54	87.1	8	12.9	7.9	0.096
Medium–high deprivation	205	171	83.4	34	16.6		
Medium deprivation	406	352	86.7	54	13.3		
Medium–low deprivation	275	250	90.9	25	9.1		
Low deprivation	96	88	91.7	8	8.3		
Employed in the health system							
No	760	632	83.2	128	16.8	51.2	0.000
Yes	294	292	99.3	2	0.7		
Connection with a nursing home							
No	877	778	88.7	99	11.3	5.3	0.022
Yes	177	146	82.5	31	17.5		
Comorbidity							
No	716	667	93.2	49	6.8	62.2	0.000
Yes	338	257	76.0	81	24.0		

Foggia province, Apulia Region, Italy

**Table 6:** Combined effects of covariates on the case vital status at the end of follow-up

Variable	OR per dead	Standard error	95% confidence interval		P > z
			Min	Max	
Age					
0–64 years (ref.)	1				
65 + years	14.61	5.45	*7.04*	*30.34*	0.000
Sex					
Women (ref.)	1				
Men	2.28	0.57	*1.39*	*3.73*	0.001
Employed in the health system					
No (ref.)	1				
Yes	0.11	0.08	*0.03*	*0.49*	0.004
Connection with a nursing home					
No (ref.)	1				
Yes	0.98	0.31	*0.53*	*1.82*	0.947
Comorbidity					
No (ref.)	1				
Yes	2.24	0.57	*1.35*	*3.69*	0.002
Constant	0.01	0.01	*0.01*	*0.03*	0.000
CT’s level of SEHDI 2011					
Var	0.50	0.64	*0.04*	*6.20*	

Foggia province, Apulia Region, Italy

*Log likelihood* = *− 275.93221; p* = *0.000*

The model in Table 6 enables a better evaluation of the impact of every covariate on the risk of death. Age was the main determinant of the risk of death (OR = 14.6; p < 0.001); moreover, being male or having at least one comorbidity significantly increased the risk of death (OR = 2.3 and 2.2, respectively; p < 0.01). On the contrary, having a connection with a nursing home was not statistically significant with regard to the risk of mortality, while being employed in the healthcare system showed a significant association with the alive status (p = 0.004).

The socio-economic status was analysed as a random effect attributed by the SEHDI of the CT of residence to each corresponding case: a statistically significant effect was found, with an increase in the probability of death with increasing deprivation. Finally, a weak statistically significant stochastic effect, expressed by the constant, was found, which increased the chances of death. The model was stable, as stated by the log-likelihood ratio and its tests (p = 0.000).

## Discussion

COVID-19 is widespread diffused, but many studies and reports worldwide have demonstrated that risk of infection and outcomes are socially patterned with the most deprived population groups usually experiencing the worst outcomes [2–14]. A narrative review of 42 studies, all focused on the first pandemic wave, found an association between socio-economic deprivation and an increased likelihood of contracting COVID-19; the strongest evidence was arisen from three large observational studies. The other compelling association was observed with regard to race or ethnicity [42]. On the other hand, a minority of studies, a number of which incidentally were conducted in Italy, did not show such association or even reported opposing results [15–20].

By using the SEHDI, our study allowed us to validly and reliably identify the main factors related to socio-economic deprivation, as demonstrated by the high total explained variance value (74.2%), and to study their impact on COVID-19 incidence and fatality.

The factors and census variables composing the SEHDI included in this analysis were the family structure, the characteristics of the house and the education level, which are known to be associated with the socio-economic status of the population [43]. In particular, the main determinants of socio-economic status considered in our study were: single-member household composition, older age, housing conditions and lower education level.

In agreement with the literature [44], most of the census variables considered in our study highlighted the discomfort of single-member families, especially those composed by an elderly individual due to their greater need for both domestic and personal care.

Regarding the housing conditions, it is well known that adequate housing and hygiene conditions are essential for preventing or limiting the spread of respiratory infections [45]. Indeed, overcrowding living conditions and poor-quality housing challenge social distancing increasing the risk of COVID-19 transmission [2, 3, 8, 22].

In terms of education level, it is well known that low education level negatively impacts health and is correlated with a higher incidence of chronical non-communicable diseases (e.g., cardiovascular diseases, diabetes, respiratory chronic diseases, cancer), which in turn may increase the susceptibility to COVID-19 infection and related mortality [25, 26, 46]. Additionally, low education is often associated with elementary occupations, which have been demonstrated to be correlated with a particularly high rate of mortality from COVID-19 [22]. Furthermore, education is recognised as a strong factor influencing health literacy and correct health behaviours both overall and in the specific COVID-19 context [47–49]. During the first epidemic wave in Italy, the already existing mortality gap between the high and low education level population groups further increased and individuals with lower education level were more exposed to those factors increasing COVID-19 mortality risk [50].

In our study, the most deprived groups lived in the capital city and the main municipalities of Foggia province, where the majority of the elderly population resided. Within the Foggia municipality, the most deprived area was the old part of the city (Southern part of the city centre), which is at a longer distance from healthcare services and has restricted availability of public transport [32]. The lower accessibility to healthcare facilities is known to influence health service utilisation and delay patients care affecting health outcomes [51, 52].

Our study showed that the majority of COVID-19 cases were aged 0–64 years, belonged to medium–low/low deprivation groups, were not employed in the healthcare system and did not have a connection with a nursing home. As for the lack of correlation between COVID-19 and high deprivation observed in our study, which is in disagreement with a part of the literature, a potential explanation behind the observed discrepancy may be the younger age of the population living in the least deprived areas of the Foggia province. Younger individuals may be more exposed to the virus due to occupational and recreational reasons. The younger age of COVID-19 cases was also confirmed by the low connection with nursing homes, the low number of resulting deaths and the low number of comorbidities in the COVID-19 case group.

Secondly, a lower ethnic heterogeneity of the Foggia population compared with most studies in the literature may have removed one of the main determinants of COVID-19 differential susceptibility (i.e., presence of racial minorities). Indeed, most studies in the literature have been conducted in the UK and US, countries with an extremely high ethnic heterogeneity [3–6, 8–10, 21]. Findings from a later phase of the pandemic confirm the different impact of deprivation and ethnicity [53].

Also the SIR distributions by sex, age and age by sex across the SEHDI groups confirmed that the incidence of COVID-19 increased with decreasing deprivation and was higher in the 0–64 year age group. Furthermore, a higher incidence of COVID-19 in men compared with women emerged from our study. Sex disparity in COVID-19 incidence and mortality has extensively been documented in the literature. Women seem to mount a stronger immune response to infections, being protected by oestrogens and being more inclined to preventive care, healthy lifestyle, and hand hygiene [54–56].

The lack of association between fatality and socio-economic status of COVID-19 cases observed in our study is in disagreement with some studies [2–14]. Compared with the other European countries, the Italian population is known to be less affected by mortality-related social inequalities thanks to the protection of the Mediterranean diet, family support and the universal character of the healthcare system [50, 57]. As opposed to our study, the similar Italian study conducted by Di Girolamo et al*.* in the Emilia Romagna region demonstrated a higher mortality burden in the most deprived areas of the region. [30]. This discrepancy could be explained by the different pattern of diffusion of COVID-19 and different COVID-19 case fatality rate during the first epidemic wave between the two Italian regions which prevent a meaningful comparison of the data. Based on the ISS-Istat report published on March 2021, 26,564 COVID-19 cases and 4238 COVID-19 deaths were reported in the Emilia Romagna region compared with 4804 cases and 537 deaths in the Apulia region during the first epidemic wave (March–May 2020) [25, 26]. During this period, the percentage of COVID-19 deaths observed in the Emilia Romagna region over the total of COVID-19-related deaths in Italy was nearly 8 times higher compared with the number reported in the Apulia region [50].

Knowledge gained as the epidemic progressed revealed that the impact of deprivation on COVID-19 outcomes has been different in the subsequent waves and varied from country to country. Studies conducted in Spain, Belgium, and Germany, observed, from the first to the last wave, an increasingly detrimental trend for the deprived subgroups or even a small advantage in terms of incidence from living in a disadvantaged area. [17, 20, 58]. These findings differ from what has been observed in the UK, where incidence during the second wave increased more in less deprived areas than in more deprived areas [59].

Multiple factors have been invoked to account for this variability, some of which, such as the diffusion of knowledge of the risk factors and the adoption of preventive behavior related to individual awareness, are inversely related to deprivation level [17, 30]. Furthermore, fake news and conspiracy theories rapidly spread online during the first wave of the COVID-19 pandemic generating confusion and insecurity among the population and potentially leading to serious implications for the individual and community [60–63]. Such misinformation regarded an exaggeration by the experts about the disease severity and the extent of virus spread, and the cause of the disease itself, among other factors [60, 61]. In such a context of misinformation, the education level appears to have contributed to health inequality as communities with lower education levels are more likely to believe in fake news leading to an increased risk of SARS-CoV-2 infection or worse disease outcome [64–67].

A study that followed the entire evolution of pandemic in the Apulia Region [19] suggests that deprivation has a greater impact the greater is the viral circulation and the fewer are the restrictions. In our study, the burden of deprivation may have been somewhat mitigated due to the fact that we addressed the first wave, when a total lockdown was imposed and when Apulia was a region with a low incidence compared to other Italian regions.

Furthermore, the lack of correlation between socio-economic status and COVID-19 fatality observed in our study may be explained with the higher incidence of COVID-19 among the least deprived population groups of the Foggia district, which may be more exposed to SARS-CoV-2 virus for occupational reasons (e.g. work-related travel to high risk areas) and lifestyle. This portion of the population resides in urban areas with prompt access to healthcare facilities and live in better conditions. Another explanation for the lack of association between fatality and socio-economic status could be the younger age of COVID-19 cases, that represents an enhancement in the survival probability.

Similarly, Gadeyne et al. reported [15], during the first COVID-19 wave in Belgium, higher excess mortality among certain subgroups, specifically high-income men aged 25–64 years, middle- and high-income men and women aged 65–84 years, and non-resident men and women aged 85 years and over. These results were ascribed to an increased risk of infection through leisure and work-related travel for the younger, affluent, and active subgroup, and to the interfamilial exposure and transmission for the older subgroup.

On the other hand, a significantly lower case fatality rate among healthcare workers (0.7% in the healthcare worker group vs. 16.8% in the non-healthcare worker group) emerged from our study. This is in line with the overall Italian trend and may be explained by more frequent testing of healthcare workers compared with the general population enabling faster detection of asymptomatic or mildly symptomatic cases [1]. A low case fatality rate among healthcare workers has also been reported worldwide by a systematic review and meta-analysis including 594 records [68].

This work has some strengths and limitations. The robust model adopted providing an accurate picture of the Foggia population by SEHDI alongside a detailed analysis of the main socio-economic, demographic and clinical factors that could have an impact on COVID-19 incidence and fatality may be considered a strength. Furthermore, the low ethnic heterogeneity of the Foggia population may have limited the weight of a determinant which was not the subject of our analysis.

A major limitation is the unavailability of the number of people tested, rather than of positive cases. It has been reported that disadvantaged people are less likely to be tested and more likely to test positive, be hospitalised or die [12, 58]. This seems to have been particularly prevalent during the first wave due to the scarcity of testing, [13, 14, 17, 58] which was reserved to symptomatic or moderate-severe cases and could represent a bias masking/underestimating the actual incidence of COVID-19 cases among the most deprived population.

Another limitation, though shared with the most studies evaluating the relation between deprivation and COVID-19 dynamics, is the adoption of a composite measure of deprivation, with the intrinsic difficulty to discriminate the role of each factor contributing to the SEHDI score.

## Conclusion

Our study did not support the hypothesis that the COVID-19 incidence and fatality burden are always heavier in the most deprived areas and provides a further contribution to the available literature whose findings are not fully consistent. Several reasons may support the reported discrepancy, including differences in population age distribution, population ethnic composition, estimation of viral circulation, COVID-19 incidence rates, and type of healthcare system. Furthermore, especially during the earlier phase of the pandemic, infection risk and outcome may be more strongly associated to behavioural aspects like social contacts and working conditions, both potentially correlated to low deprivation patterns. Further research is needed to better understand the correlation between COVID-19 incidence/mortality and socio-economic status and identify the underlying mechanisms. This better understanding could help adapt and tailor preventive strategies according to the vulnerability of certain population subgroups in order to reduce social and healthcare inequalities generated by the pandemic.

## Data Availability

The authors declare that the data supporting the findings of this study are available within the article and upon reasonable request and with permission of Apulian Public Health Authority.

## Abbreviations

ANOVA: Analysis of variance
COVID-19: Coronavirus Disease 2019
CS: Census Tract
GIS: Geographic information system
OR: Odds ratio
SARS-CoV-2: Severe Acute Respiratory Syndrome coronavirus 2
SES: Socio-economic status
SEHDI: Socio-Economic and Health-related Deprivation Index
SIR: Standardised Incidence Ratio
